# A multi-omics approach reveals dysregulated TNF-related signaling pathways in circulating NK and T cell subsets of young children with autism

**DOI:** 10.1038/s41435-025-00349-z

**Published:** 2025-07-30

**Authors:** Wared Nour-Eldine, Samia M. Ltaief, Khalid Ouararhni, Nimshitha P. Abdul Manaph, Alberto de la Fuente, Ilham Bensmail, Houari B. Abdesselem, Abeer R. Al-Shammari

**Affiliations:** 1 Neurological Disorders Research Center, Qatar Biomedical Research Institute, Hamad Bin Khalifa University, Qatar Foundation, Doha, Qatar; 2 Genomics Core Facility, Qatar Biomedical Research Institute, Hamad Bin Khalifa University, Qatar Foundation, Doha, Qatar; 3 Diabetes Research Center, Qatar Biomedical Research Institute, Hamad Bin Khalifa University, Qatar Foundation, Doha, Qatar; 4 Proteomics Core Facility, Qatar Biomedical Research Institute, Hamad Bin Khalifa University, Qatar Foundation, Doha, Qatar; 5 College of Health and Life Sciences, Hamad Bin Khalifa University, Qatar Foundation, Doha, Qatar

**Keywords:** Gene expression, Lymphocytes

## Abstract

Peripheral immune dysregulation is frequently reported in autism spectrum disorder (ASD); however, the underlying molecular mechanisms remain unclear. We recruited a well-defined cohort of young Arab children with ASD, aged 2–4 years, along with matched controls in Qatar. Using a multimodal approach, we integrated transcriptomic, proteomic, and single-cell RNA-seq data analyses from this cohort. Targeted transcriptomic profiling identified differential expression of 50 immune-related genes in the circulating PBMCs of children with ASD, three of which (JAK3, CUL2, and CARD11) negatively correlated with ASD symptom severity. These gene signatures were validated in independent studies using blood and brain tissues from individuals with ASD. Enrichment analysis revealed involvement of these genes in immune function, particularly through TNF signaling pathway. Proteomic analysis highlighted disrupted TNF signaling and upregulated levels of TNFSF10 (TRAIL), TNFSF11 (RANKL), and TNFSF12 (TWEAK) in plasma of individuals with ASD. Single-cell RNA-seq revealed that B cells, CD4 T cells, and NK cells potentially contributed to these upregulations in ASD. Dysregulated TRAIL, RANKL, and TWEAK signaling pathways were specifically observed in CD8 T cells, CD4 T cells, and NK cells of individuals with ASD. These findings provide new insights into immune dysregulation mechanisms in ASD and highlight potential therapeutic targets.

## Introduction

Autism spectrum disorder (ASD) is a heterogeneous neurodevelopmental disorder characterized by challenges in social interactions and communication as well as repetitive behaviors and interests. Generally, a child with ASD first shows symptoms during the first three years of life. However, the clinical severity of ASD symptoms is largely variable among affected individuals, which creates challenges for accurate and early diagnosis of ASD. The prevalence of ASD is estimated to be 1 in 100 children according to the World Health Organization epidemiological data (2023) [1]; however, the etiology remains unclear, but likely involves genetic and environmental factors and complex interactions between them [2]. Current evidence supports a link between the immune system and ASD development. Previous studies have shown that several genes associated with ASD are immune-related genes, and the majority of the environmental risk factors associated with this disorder, such as toxicants and infectious agents, are also known to trigger immune responses [3–5].

Immune abnormalities and inflammation are observed in the peripheral blood of individuals with ASD [6–8]. Immune conditions, such as allergy, asthma, eczema, and gastrointestinal complications, are more common in individuals with ASD than in the general population [9]. Furthermore, numerous studies have reported abnormal cytokine levels and dysregulated functioning of immune cells in the peripheral blood of subjects with ASD, which are also associated with an increased severity of ASD symptoms [7, 8]. For example, abnormal levels of several cytokines, such as interleukin (IL)-6, tumor necrosis factor (TNF)-α, and IL-1β, have been reported in the blood of subjects with ASD, which are associated with worse behavioral outcomes [10–14]. Similarly, disrupted functions of peripheral immune cells, such as T cells, monocytes, and natural killer (NK) cells, have also been reported in ASD [12, 15–19]. Importantly, increased cytokine levels in the peripheral blood of subjects with ASD are also associated with similar elevations in cytokine levels in the brain, which could ultimately lead to disrupted synaptic plasticity and impaired behavioral outcomes [3, 20, 21]. Therefore, immune aberrations in the peripheral blood of individuals with ASD may reflect immune changes in the brain and behavioral outcomes.

Despite numerous reports of altered immune phenotypes in the peripheral immune system in ASD, the underlying molecular mechanisms that may lead to this dysfunction remain unclear. In this study, we recruited a well-characterized study cohort of young Arab children with ASD, aged 2–4 years, along with their matched control subjects from the Qatari population. We conducted a comprehensive approach by combining transcriptomics, proteomics, and single-cell RNA sequencing data analyses from the same study cohort. This strategy could provide novel insights into dysregulated immune signaling pathways in ASD, ranging from general immune cells and molecules in circulation to specific cell types within ASD. These findings could pave the way for future research aimed at understanding the mechanisms behind these dysregulations, potentially identifying new therapeutic targets for ASD.

## Materials and methods

### Study population

The study was reviewed and approved by the Institutional Review Board (IRB) Ethics Committee of Hamad Medical Corporation (HMC, Doha, Qatar; approval number: MRC-02-18-116) for the recruitment of subjects with ASD and the Primary Health Care Corporation (PHCC, Doha, Qatar; approval number: 2020/06/064) for the recruitment of control subjects. This study also received exempt IRB approval from the Qatar Biomedical Research Institute (QBRI, Doha, Qatar; approval number: 2019-003). This study was conducted in accordance with the guidelines of the Declaration of Helsinki. Written informed consent for study participation was obtained from the families of all subjects. Participants with ASD were recruited from the Child Development Center in Rumailah Hospital of HMC, and their matched control participants were recruited from Al-Wajbah Health Center of PHCC.

### Subject screening and enrollment in the study

All participants were initially screened using a questionnaire to confirm their adherence to our study’s eligibility criteria as previously described [22, 23]. The subjects enrolled in this study were of Arabic ethnicity and between the ages of 2 and 4 years. Additionally, all subjects fulfilled the following criteria: (1) being born in Qatar; (2) having their mothers reside in Qatar for most of their pregnancy; (3) primarily living in Qatar since birth; (4) absence of immune conditions like autoimmune diseases, asthma, allergies, and eczema; (5) no neurological conditions such as epilepsy; (6) no suspected difficulties with vision, hearing, or mobility; (7) no other medical complications such as heart, lung, or kidney diseases; and (8) not taking medications or having recent infections or vaccinations at the time of study enrollment. Control subjects were also required to have no family history of ASD and were assessed using the Social Communication Questionnaire (lifetime version) with a cutoff score < 12 to eliminate ASD risk. Those diagnosed with ASD had received a clinical confirmation by qualified professionals according to the Diagnostic and Statistical Manual of Mental Disorders (DSM-5) and Autism Diagnostic Observation Schedule-second edition (ADOS-2).

### Sample collection and processing

Blood samples were extracted from the enrolled subjects into EDTA-containing anti-coagulant tubes (cat #3366643; BD Diagnostics, USA) at HMC or PHCC sites, transported, and processed in the research facility at QBRI within two hours of sample collection. Blood samples were slowly layered over Histopaque-1077 (cat #10771; Sigma-Aldrich, UK) at an equal ratio and centrifuged at 400 × *g* for 30 min with an acceleration of 3 and zero braking at standard room temperature of 20–23 °C. After centrifugation, plasma samples were collected from the upper layer into a new tube while the peripheral blood mononuclear cells (PBMCs) were carefully collected from the middle fraction into another tube without mixing with the plasma collected from the upper layer. The cells were washed twice with 1x PBS (cat #10010015; Thermo Fisher Scientific, UK), centrifuged at 400 × *g* for 10 min, then cryopreserved at −150 °C in liquid nitrogen for later use. Plasma samples were centrifuged at ~1800 × *g* for 15 min to remove cell debris, and plasma aliquots of 200 μl were stored at –80 °C until further analysis.

### RNA isolation

RNA was extracted from frozen PBMCs (protocol #MAN-10051-05; NanoString Technologies, Seattle, WA, USA), according to the manufacturer’s instructions. We used the Invitrogen Purelink RNA kit (cat #12183018 A; Thermo Fisher Scientific, USA) and eluted the RNA samples in 30 μL RNAse-DNase free water (cat #10977-015; Thermo Fisher Scientific, UK). A NanoDrop 2000/2000c Spectrophotometer (cat #ND-2000; Thermo Fisher Scientific, USA) was used to perform RNA quality checks, which showed a 260/280 ratio of approximately 1.7–2.0. The samples were stored at −80 °C until further use in downstream analysis.

### NanoString nCounter assay

The nCounter Human Immune Exhaustion panel consisting of 785 target genes (cat #XT-H-EXHAUST-12; NanoString Technologies) and the nCounter Master kit (cat #NAA-AKIT-012; NanoString Technologies) were used to perform RNA hybridization experiments (protocol #MAN-10056-05; NanoString Technologies) according to the manufacturer’s instructions. Each sample contained 100 ng of RNA. All hybridization reactions were 16 h long, in which the PrepStation runs were held in high-sensitivity mode and the data were gathered by scanning the prepared samples using the nCounter Digital Analyzer. Quality checks, data normalization, and advanced analysis modules were conducted using the Rosalind platform for nCounter Analysis according to the manufacturer’s instructions. All samples passed the quality check (QC), and no QC flags were detected. Raw data of each sample were normalized to the positive and negative controls as well as housekeeping genes included in the panel using the geNORM algorithm implemented in the Rosalind platform. Advanced Analysis tools were used to conduct differential expression analysis across the different study groups. *P*-value adjustment was performed using the Benjamini-Hochberg method of estimating false discovery rates (FDR). Significance was determined at a cutoff of absolute fold change of 1.25 and adjusted *p* < 0.05.

### BaseSpace Correlation Engine

BaseSpace Correlation Engine (BSCE; Illumina) was used to validate our gene signatures identified through nCounter assays by comparing our gene list across independent ASD studies using blood and brain samples. Additionally, BSCE was used to perform pathway enrichment analysis for the differentially expressed genes. The BSCE platform accommodates large-scale genomic, epigenetic, proteomic, and assay data. It integrates data from global public repositories and leverages advanced data-driven techniques, such as correlation analysis, aggregation, and machine learning, to process omics data using standardized pipelines specific to the platform. BSCE includes curated bioset lists consisting of high-quality, statistically validated genes, with each gene from RNA studies meeting a minimum absolute fold change of 1.2 and a *p* < 0.05. The platform utilizes an Illumina-developed Running Fisher algorithm, which compares an imported query bioset to a target bioset, analogous to the Gene Set Enrichment Analysis (GSEA) method. By employing rank-based statistical methods, BSCE calculates correlations between biosets in the imported data and those curated by the platform. Ultimately, BSCE provides researchers with the opportunity to place their experimental findings within the broader context of expertly curated omics data, which could facilitate the discovery of novel associations, the design of new experiments, and the validation of their results through independent datasets.

### Sample preparation for single-cell RNA sequencing

We have used Chromium Next GEM Single Cell 3′ GEM v3.1 Reagent Kit (10X Genomics) to prepare the PBMC samples for single-cell RNA sequencing according to the manufacturer’s instructions. Briefly, frozen PBMCs were rapidly thawed in 37 °C water bath for 2 min, and the vials were removed when a tiny ice crystal was left. Thawed PBMCs were rinsed with 1 ml pre-warmed complete growth media of RPMI supplemented with 10% FBS, then transferred to 15 ml tube containing 9 ml complete growth media. Cells were centrifuged at 300 × *g* for 5 min at room temperature, removed the supernatant, and resuspended the cell pellets in 1x PBS with 0.04% BSA. Cell concentrations were determined using automated cell counter (TC20, Bio-Rad) and the samples were resuspended at a viable cell concentration of 700–1200 cells/μl then proceeded immediately with single-cell RNA sequencing protocols. These protocols included GEM generation and barcoding, cDNA amplification, and library construction and sequencing according to the manufacturer’s instructions (10X Genomics).

### Bioinformatics analysis of single-cell RNA data

Raw base call files (BCLs) generated by Illumina NovaSeq 600 sequencer were demultiplexed using cellranger mkfastq (version 7.2.0). The FASTQ files obtained were then processed through Cell Ranger-Gene expression tool implemented in Partek Flow software (version 10.0). Briefly, the reads were trimmed and aligned to human reference genome hg38 (GRCh38), then deduplicated to obtain one alignment per unique molecular identifier (UMI), choosing Ensemble Transcripts release 104 for the annotation. We then filtered out barcodes associated with droplets containing no cells and the aligned reads were quantified to generate a single cell count matrix. Next, we downloaded the R script from Seurat’s Azimuth web-based application for human PBMCs to perform subsequent analysis in R software (R version 4.3.3) [24]. We applied filtering steps to exclude doublets and low-quality cells based on the following criteria: (1) detected genes fewer than 300 or greater than 2500 genes; (2) UMI counts fewer than 500 or greater than 20,000 counts; and (3) mitochondrial percentages greater than 15%. Doublets were further identified and removed using DoubletFinder function as previously published [25]. After stringent QC filtering steps, 54,377 cells were retained for downstream analysis. The filtered counts were processed according to the analysis pipeline from Azimuth’s reference-based mapping for human PBMCs [24]. This included data normalization using the SCTransform function and cell annotations (celltype.l1) with the FindTransferAnchors, TransferData, and IntegrateEmbeddings functions as previously described [24].

Single-cell RNA-seq data were analyzed using iDEP platform [26]. Read counts for each cell type were uploaded into iDEP (version 2.01), and data were transformed using the EdgeR tool. Differential expression analysis was performed using the limma-voom method while adjusting for age and sex. Data were considered statistically significant when the FDR was < 0.05 with a minimum fold change of 1.5. Additionally, single-cell RNA data were used to calculate the inflammation score for each cell type as previously described [27]. We obtained a list of 200 genes corresponding to the Hallmark Inflammatory Response from the Molecular Signatures Database (MSigDB), and the scores were calculated in R software [27, 28].

### Olink proteomic assays

Plasma samples were analyzed using two Olink Target Panels focused on inflammation and immune response, commercially available from Olink (Uppsala, Sweden). Each panel contains a total of 92 protein targets. Samples were analyzed according to Olink’s standard protocol. Briefly, the plasma samples were randomized and aliquoted into 96-well plates. Each sample was incubated with a pool of oligonucleotide-labeled antibody pairs that bind to specific target proteins. In this configuration, proximity-dependent DNA polymerization occurs, and the resulting amplicons were quantified using a microfluidic chip-based qPCR system on a Fluidigm Biomark instrument. The signal from each unique DNA amplicon is proportional to the protein concentration and is expressed as a normalized protein expression (NPX) value. Various internal controls were included in each run to minimize inter- and intra-assay variability. The runs were conducted at QBRI in an Olink-certified proteomics core facility. All samples passed QC assessment and were used for downstream analysis. Differential expression analysis of Olink data was performed using the iDEP platform, version 0.96 [26]. Olink NPX values were uploaded into iDEP software, and we found that covariate adjustment for age and sex did not affect the results. Statistical significance was determined using an FDR of 0.05 and a fold change cutoff of 1.25.

### Statistical analysis

Demographic data were tested for normality using the Shapiro–Wilk test. Data were analyzed with the chi-square test for categorical variables and the Mann–Whitney *U* test for continuous variables in SPSS (version 26.0). Correlation analysis was performed using Pearson’s correlation coefficients for continuous variables in GraphPad Prism (version 10). Data were considered statistically significant when the *p*-value was < 0.05.

## Results

### Study design and demographics of the study population

In this study, we carefully selected our study population to ensure that participants met our eligibility criteria, as detailed in the Methods section. This approach was designed to limit potential confounding factors, such as age differences, ethnicity, and comorbid immune conditions, which could influence the results. We also ensured that the mothers of all enrolled subjects had lived in Qatar for the majority of their pregnancies, that the subjects were born in Qatar, and that they continued to live primarily in Qatar until the time of study participation. This criterion aims to control for the external environment to which the subjects were exposed during prenatal and postnatal development.

The study population comprised of children with ASD and control subjects. For nCounter assays, we included a total of 72 children (*n* = 48 ASD, *n* = 24 control) as summarized in Table 1. The study population showed matching demographic characteristics, in terms of age, sex, and nationality (Table 1). The age of children with ASD [median (interquartile range, IQR)] was 3.37 (3.02–3.67) years, which was comparable to the age of the control subjects 3.46 (2.82–3.71) years (*p* = 0.895, Table 1). Additionally, the two study groups were sex-matched, with male-to-female ratios of 85.4/14.6 and 83.3/16.7 in the ASD and control groups, respectively (*p* = 0.535, Table 1). Furthermore, both study groups showed matching distribution of three Arab nationalities, namely Qatari (50 vs. 41.7), Egyptian (25 vs. 29.2), and Syrian (25 vs. 29.2), in the ASD and control groups, respectively (*p* = 0.800, Table 1).

**Table 1:** Demographic characteristics of the study population.

Characteristic	Categories	Total (*n* = 72)	Control (*n* = 24)	ASD (*n* = 48)	*p*-value
Age					
	Age in years	3.37 (2.97–3.69)	3.46 (2.82–3.71)	3.37 (3.02–3.67)	0.895
Sex					
	Male	61(84.7)	20 (83.3)	41 (85.4)	0.535
	Female	11 (15.3)	4 (16.7)	7 (14.6)	
Family history of ASD					
	Yes	–	N/A	10 (20.8)	–
	No	–	N/A	38 (79.2)	
Consanguinity					
	Yes	21 (29.2)	10 (41.7)	11 (22.9)	0.099
	No	51 (70.8)	14 (58.3)	37 (77.1)	
Method of reproduction					
	Natural	69 (95.8)	23 (95.8)	46 (95.8)	0.710
	Assisted (IVF)	3 (4.2)	1 (4.2)	2 (4.2)	
Maternal complications					
	Yes	27 (37.5)	7 (29.2)	20 (41.7)	0.302
	Diabetes	18 (25)	4 (16.7)	14 (29.2)	
	Hypertension	2 (2.8)	0 (0)	2 (4.2)	
	Allergy	2 (2.8)	0 (0)	2 (4.2)	
	Multiple conditions (asthma, allergy, diabetes, and/or hypertension)	4 (5.6)	3 (12.5)	1 (2.1)	
	No	45 (62.5)	17 (70.8)	28 (58.3)	
Pregnancy duration					
	< 9 months	4 (5.6)	2 (8.3)	2 (4.2)	0.407
	≥ 9 months	68 (94.4)	22 (91.7)	46 (95.8)	
Maternal age at labor					
	Age in years	31.00 (23.00–42.00)	31.00 (23.00–40.00)	30.05 (23.00–42.00)	0.747
	< 35 years	53 (73.6)	20 (83.8)	33 (68.8)	0.189
	≥ 35 years	19 (26.4)	4 (16.7)	15 (31.3)	
Type of delivery					
	Normal	40 (55.6)	12 (50)	28 (58.3)	0.531
	C-section	27 (37.5)	11 (45.8)	16 (33.3)	
	Induced	5 (6.9)	1 (4.2)	4 (8.3)	
Postnatal complications					
	Yes	10 (13.9)	3 (12.5)	7 (14.6)	0.559
	Hypoxia	8 (11.1)	2 (8.3)	6 (12.5)	
	Jaundice	0 (0.0)	0 (0.0)	0 (0.0)	
	Hypoxia and Jaundice	1 (1.4)	0 (0.0)	1 (2.1)	
	Others	1 (1.4)	1 (4.2)	0 (0.0)	
	No	62 (86.1)	21 (87.5)	41 (85.4)	
Birth weight					
	Weight in kg	3.01 (1.70–4.50)	3.00 (1.87–3.76)	3.06 (1.70–4.50)	0.990
	< 2.5 kg	10 (13.9)	3 (12.5)	7 (14.6)	0.559
	≥ 2.5 kg	62 (86.1)	21 (87.5)	41 (85.4)	
Nationality					
	Qatari	34 (47.2)	10 (41.7)	24 (50)	0.800
	Egyptian	19 (26.4)	7 (29.2)	12 (25)	
	Syrian	19 (26.4)	7 (29.2)	12 (25)	

Data are presented as medians (lower–upper quartile) or *n* (%).

*P*-values were assessed by the Mann–Whitney *U* test for continuous variables and Pearson’s chi-square or Fisher’s exact test as appropriate for categorical variables. N/A: not applicable.

Meanwhile, there were no differences between children with ASD and controls in terms of consanguinity, method of reproduction (natural or assisted), maternal complications (diabetes, hypertension, asthma, or allergy), pregnancy duration, maternal age at delivery, type of delivery (normal, c-section, or induced), postnatal complications (hypoxia or jaundice), or birth weight (Table 1). For single-cell RNA sequencing and Olink experiments, we selected a total of 34 children from the aforementioned population, including 23 children with ASD (21 males, 2 females) and 11 controls (9 males, 2 females), all of whom were Qatari and aged 2–4 years, as described earlier.

### Altered expression of immune genes in ASD and their association with ASD severity

**Fig. 1:**
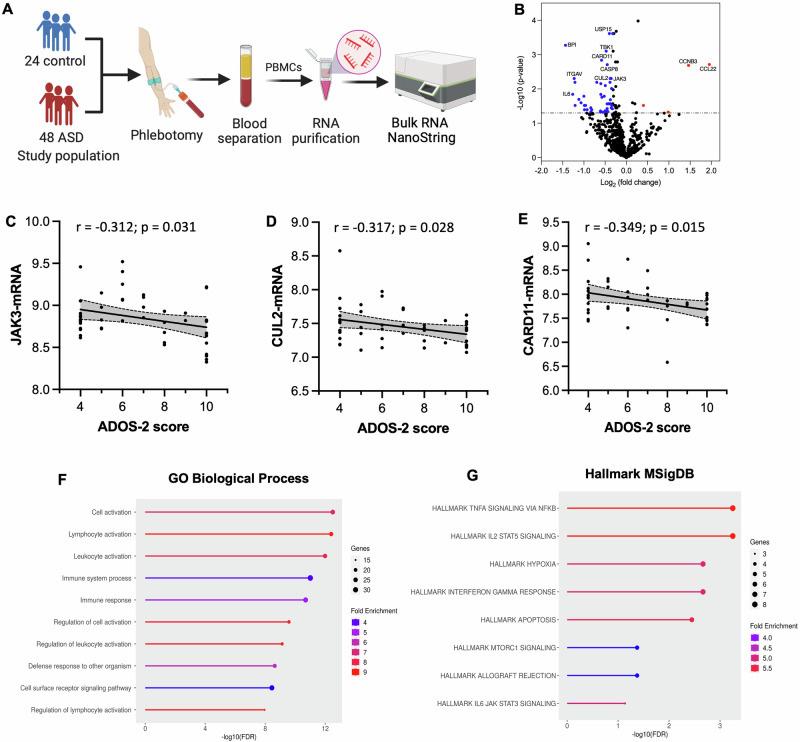
Differential expression of immune genes in PBMCs of children with ASD. **A** Schematic diagram illustrating the experimental design. **B** Volcano plot showing 4 upregulated and 46 downregulated genes in ASD vs. control group. **C**–**E** Scatter plots demonstrating negative correlations between JAK3, CUL2, and CARD11 genes and the severity level of ASD symptoms. **F**–**G** Biclustering analysis of the 50 DEGs shows enrichment in several immune pathways based on the GO Biological Process and Hallmark MSigDB databases.

We utilized nCounter immune exhaustion panel to conduct a targeted transcriptomic analysis of immune genes in circulating PBMCs from individuals with ASD (*n* = 48) compared to the control group (*n* = 24), as illustrated in Fig. 1A. The full list of genes and probe details was downloaded from the manufacturer’s website and included in Supplementary Table 1. We detected differential expression of several genes as displayed in the volcano plot in Fig. 1B. Specifically, we found 50 differentially expressed genes (DEGs), in which 4 genes were significantly upregulated, and 46 genes were significantly downregulated in the ASD vs. control group as listed in Supplementary Table 2. Notably, these DEGs are associated with several signaling pathways and functions as highlighted in Supplementary Table 1. Besides, we analyzed the correlation between the identified genes and ASD severity, and we found negative correlations between ADOS-2 scores and JAK3 (*r* = −0.312, *p* = 0.031), CUL2 (*r* = −0.317, *p* = 0.028), and CARD11(*r* = −0.349, *p* = 0.015) genes (Fig. 1C–E).

### Validation of our gene signatures in independent ASD studies

We next used the BSCE platform to validate our list of 50 DEGs across several independent studies in ASD. We analyzed the overlap between our dataset and six independent ASD studies that were conducted using human blood samples (2 studies), human brain tissues (1 study), and mouse brain tissues (3 studies).

Interestingly, we identified a total of 18 common genes between our dataset and two other studies on human blood samples in ASD, namely CYP4A11, TRIM29, PIK3R3, ZBTB16, CIITA, CARD11, CAMK2D, RORA, NFATC2, CCL5, CREBBP, ZFP36L2, NFKB2, JAK3, ITCH, INPP5D, CASP8, and FAS (Supplementary Fig. 1 and Supplementary Table 3) [29, 30]. Interestingly, most of these genes were positively correlated with our dataset. We then explored whether our list of DEGs in blood samples was also correlated with differential expression in brain samples of human subjects with ASD and mouse models of the disorder. Remarkably, several genes from our dataset were differentially expressed in brain tissues in ASD, and many of them showed positive correlations with our dataset. In particular, we found a total of 21 common genes between our dataset and another study conducted on the prefrontal cortex of human subjects with ASD, namely CCL22, CCNB3, IL6, PDGFRA, ASB4, STAT4, RORA, CASP8, DIABLO, SLC2A14, TRIM29, PTPRK, ZBTB16, CAMK2D, TELO2, TBK1, BCL2, CREBBP, ZFP36L2, JAK3, and ITCH (Supplementary Fig. 2 and Supplementary Table 3) [31]. In addition, we found a total of 28 common genes between our dataset and three other studies conducted on the mouse primary cortical neurons with genetic knockdown of several candidate genes associated with ASD, namely NLGN1, NLGN3, MeCP2, and SHANK3 (Supplementary Fig. 3) [32–34]. The list of these common genes is included in Supplementary Table 3. Overall, we found a total of 41 common genes among both our dataset and independent studies on the human blood samples, brain tissues of human subjects and mouse models of the disorder as listed in Supplementary Table 3.

### Molecular functions of altered gene signatures in ASD

We then explored the functional roles of altered gene signatures in ASD. First, we conducted a biclustering analysis of the 50 DEGs using the iDEP platform to find the most affected signaling pathways in ASD. Our analysis revealed enrichment of several immune pathways in ASD, including lymphocyte activation, immune response, and cell surface receptor signaling pathway (Fig. 1F). In particular, we identified TNF-alpha signaling via NFkB as the major pathway enriched in the ASD vs. control group, along with other pathways such as IL2-STAT5 signaling and the interferon-gamma response (Fig. 1G). These findings are also supported by nCounter functional annotations of the 50 DEGs (Supplementary Table 1). Next, we sought to determine the direction of change for these signaling pathways–whether they are upregulated or downregulated in ASD. Hence, we conducted pathway enrichment analysis using the BSCE platform, which revealed a significant downregulation of lymphocyte activation (*p* = 3.10E-20) and immune response-activating signal transduction (*p* = 5.40E-12) in the ASD vs. control group (Supplementary Table 4). Additionally, we observed significant downregulation of TNF-alpha signaling via NFkB (*p* = 3.50E-12), IL2-STAT5-signaling (*p* = 4.20E-12) and the interferon-gamma response (*p* = 2.40E-11) in the ASD vs. control group (Supplementary Table 4). These findings suggest impaired immune functions in ASD, particularly linked to disrupted TNF-alpha signaling pathway.

### Global disruption of inflammation in several immune cell types in ASD

**Fig. 2:**
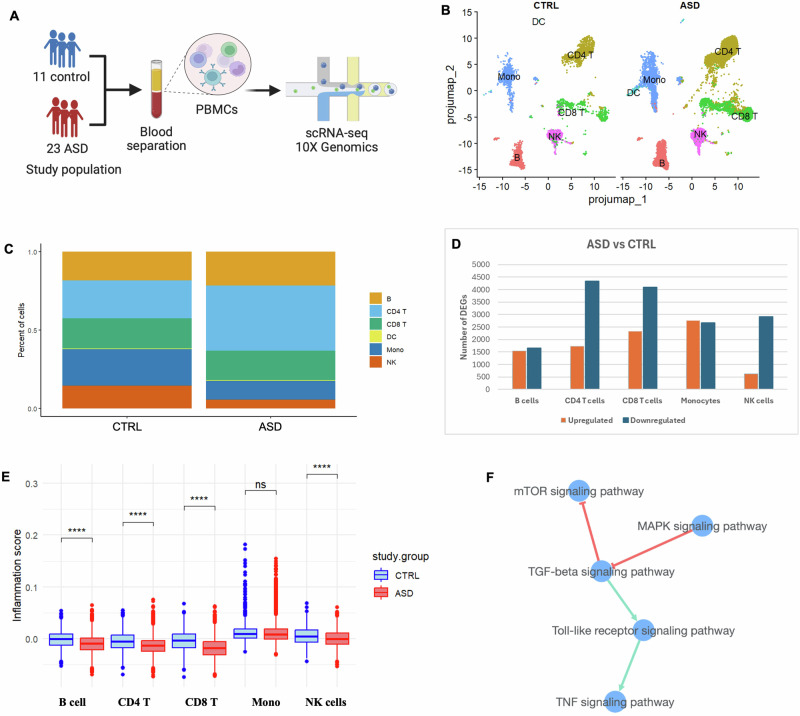
Single-cell RNA analysis of PBMCs reveals a global disruption of inflammation across several immune cell types in ASD. **A** Graphical representation of the experimental design. **B** UMAP projections of ASD and control samples labeled according to Azimuth reference-based mapping for human PBMCs. **C** Barplots showing the representative proportions of the classified cell types separated by the study condition. **D** Barplots showing the number of upregulated and downregulated genes in the classified cell populations in ASD vs. control group. **E** Boxplots showing overall reduced inflammation scores in several immune cells in ASD (red) compared to the control group (blue). **F** Pathway crosstalk analysis in the XTalkDB database reveals interactions between five signaling pathways, including the TNF signaling pathway. ****P < 0.0001; ns: non-significant.

We then sought to investigate which specific immune cell types in ASD exhibit disrupted immune phenotypes at the single cell level. To this end, we conducted single-cell RNA sequencing analysis on a total of 34 randomly selected PBMC samples from the same study cohort. These included samples from 11 control and 23 ASD subjects, all of whom were from Qatari children matched for age and sex (Fig. 2A). We identified the five major peripheral immune cells, namely NK cells, monocytes, CD4 T, CD8 T, and B cells using reference-based mapping for human PBMCs with the Azimuth tool (Fig. 2B). The percentage of each cell type in the two study groups is shown in Fig. 2C. Notably, CD4 T cells were more abundant in the ASD group, whereas monocytes and NK cells had reduced percentages in ASD compared to the control group, although the observed tendency did not reach statistical significance (Fig. 2C).

Interestingly, we identified thousands of cell type-specific DEGs between ASD and control groups, with CD8 T cells exhibiting the highest number of DEGs compared to other cell types. The full list of DEGs and the total number of upregulated and downregulated genes for each cell type are included in Fig. 2D and Supplementary Table 5. To gain insights into the inflammatory response in ASD, we analyzed the expression of a set of 200 genes linked to inflammation and calculated an inflammation score for each cell type, as previously described [35]. We observed significant reduction in the inflammation score specifically in B cells, CD4 T, CD8 T, and NK cells, but not in monocytes, in the ASD vs. control group (*p* < 0.0001; Fig. 2E).

We then sought to determine the crosstalk between immune signaling pathways that may contribute to the impaired inflammatory response in ASD. As previously described, the list of 50 DEGs we identified in ASD are associated with several signaling pathways, according to nCounter annotation (Supplementary Table 1). To explore this further, we conducted a pathway crosstalk analysis using the XTalkDB database and we filtered for data curated specifically for humans and PBMC immune subtypes relevant to this study [36]. This analysis revealed interactions between five signaling pathways, including TNF signaling pathway, as shown in Fig. 2F. Overall, these findings suggest the presence of an inhibited inflammatory response in several immune subtypes in ASD, potentially due to dysregulated crosstalk between multiple immune signaling pathways, including the TNF signaling pathway.

### Abundance of circulating proteins involved in inflammation and immune response in ASD

**Fig. 3:**
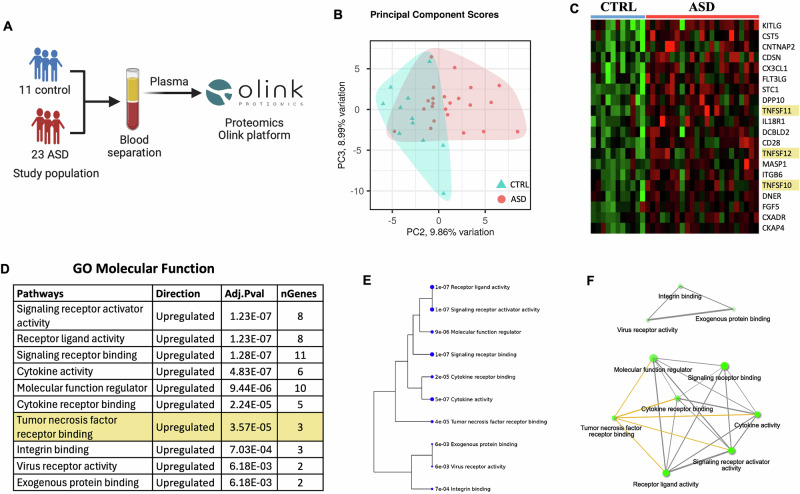
Proteomics analysis of plasma samples demonstrates upregulated levels of several proteins involved in inflammation and immune response in ASD. **A** Schematic diagram for the experimental design. **B** Principal component analysis shows the separation between ASD and control groups based on the expression profiles of proteins. **C** Heatmap displaying differentially expressed proteins across individual samples in ASD and control groups (color gradient: green “low expression” to red “high expression”). **D** List of the enriched pathways in ASD vs. control group, which includes tumor necrosis factor receptor binding (highlighted). **E**, **F** Enrichment tree and network analysis demonstrating the link between the dysregulated pathways in ASD.

We next analyzed the levels of circulating proteins associated with inflammation and immune response to determine whether they were altered in ASD. Olink proteomic analysis was performed on plasma samples from the same subjects previously selected for single-cell RNA analysis (Fig. 3A). Principal component (PC) analysis showed clear separation between ASD and control groups based on their proteomic expression profiles, with PC2 significantly correlated with the group classification (*p* = 2.36E-03, Fig. 3B). Overall, we identified 20 differentially expressed proteins between the ASD and control groups as shown in Fig. 3C and Supplementary Table 6. Pathway enrichment and network analyses revealed upregulation of several signaling pathways in ASD, which were also linked to each other, as demonstrated in Fig. 3D–F. Of particular interest, we found upregulation of the TNF receptor binding pathway in ASD (*p* = 3.57E-05), which we had previously identified in this study as the top pathway impacted in ASD based on transcriptomic analyses of PBMC samples. This signaling pathway in ASD was primarily driven by the increased expression of three circulating molecules—TNFSF10, TNFSF11, and TNFSF12—which are involved in the TRAIL, RANKL, and TWEAK signaling pathways, respectively. Therefore, we subsequently focused on these three signaling pathways to determine how they are impacted at the single-cell level in ASD.

### Disrupted TRAIL, RANKL, and TWEAK signaling pathways in specific immune cell types in ASD

**Fig. 4:**
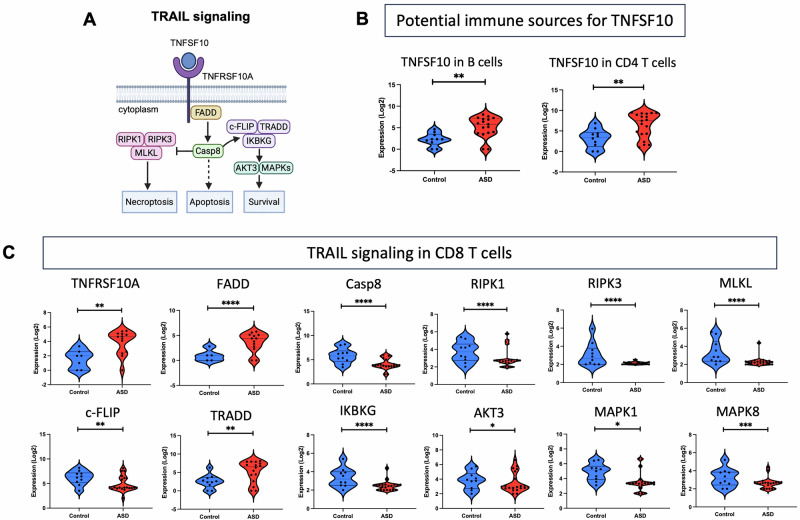
Dysregulated TRAIL signaling pathway in CD8 T cells of children with ASD. **A** Schematic diagrams illustrating key molecules and the link between them in TRAIL signaling pathway. **B** Violin plots showing potential immune sources for upregulated TNFSF10 in the plasma in ASD. **C** Violin plots demonstrating dysregulated expression of key molecules in TRAIL pathway in CD8 T cells of individuals with ASD. *P < 0.05; **P < 0.01; ***P < 0.001; ****P < 0.0001.

**Fig. 5:**
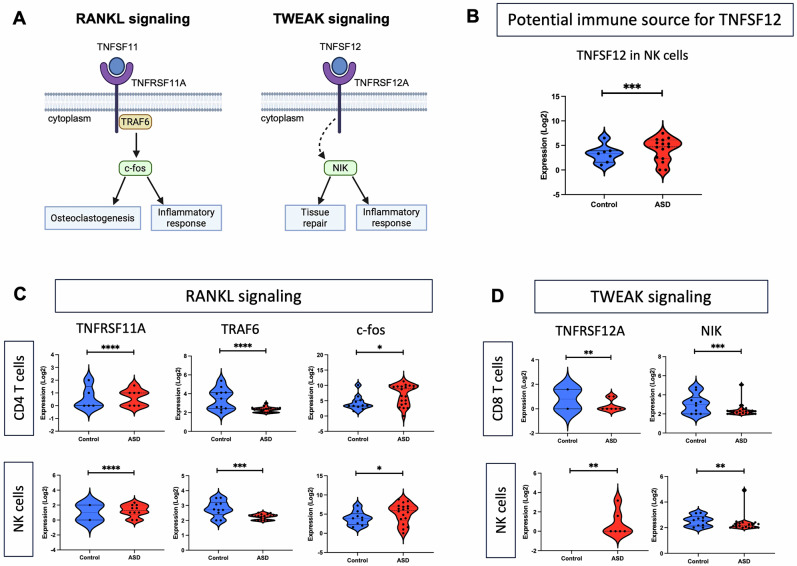
Disrupted RANKL and TWEAK signaling pathways in cell-type specific immune cells in ASD. **A** Schematic diagrams illustrating key molecules and the link between them in RANKL and TWEAK signaling pathways. **B** Violin plots showing potential immune source for upregulated TNFSF12 in the plasma in ASD. **C–D** Violin plots demonstrating dysregulated expression of key molecules in RANKL and TWEAK pathways in specific immune cell-types in ASD. *P < 0.05; **P < 0.01; ***P < 0.001; ****P < 0.0001.

We performed single-cell RNA analysis to explore whether specific immune cells in ASD may contribute to the upregulated levels of circulating TNFSF10–12 molecules, and whether these cell types exhibit dysregulated signaling associated with TNFSF10–12 pathways. Hence, we analyzed the expression of several key molecules in TNFSF10 (TRAIL), TNFSF11 (RANKL), and TNFSF12 (TWEAK) signaling pathways as listed in Table 2 and illustrated in Figs. 4A and 5A. Remarkably, TNFSF10 was significantly upregulated in B and CD4 T cells, suggesting that these two cell types might be potential sources for the upregulated plasma levels of TNFSF10 in ASD (Table 2 and Fig. 4B). Additionally, we found significant dysregulation of several TRAIL signaling molecules specifically in CD8 T cells from children with ASD, including TNFRSF10A, FADD, Casp8, RIPK1, RIPK3, MLKL, c-FLIP, TRADD, IKBKG, AKT3, MAPK1, and MAPK8, as shown in Table 2 and Fig. 4C.

**Table 2:** List of differentially expressed genes belonging to TRAIL, RANKL, and TWEAK signaling pathways in specific immune cell types in ASD.

Sources/Pathways	Cell type	Gene name	Fold change	Adjusted p-value
Immune sources for TNFSF10 & TNFSF12	B cells	TNFSF10	1.789	0.002
	CD4 T cells	TNFSF10	1.609	0.003
	NK cells	TNFSF12	2.253	1.00E-04
TRAIL signaling				
	CD8 T cells	TNFRSF10A	1.584	0.004
		FADD	2.333	6.23E-06
		Casp8	–2.150	1.25E-05
		RIPK1	−1.857	5.11E-05
		RIPK3	−2.587	4.77E-06
		MLKL	−2.820	5.72E-08
		c-FLIP	−1.554	0.003
		TRADD	1.809	0.003
		IKBKG	−1.685	2.43E-05
		AKT3	−1.505	0.041
		MAPK1	−1.553	0.017
		MAPK8	−1.546	6.59E-04
RANKL signaling				
	CD4 T cells	TNFRSF11A	−6.024	3.63E-08
		TRAF6	−1.735	3.12E-05
		c-fos	1.839	0.044
	NK cells	TNFRSF11A	3.396	4.18E-05
		TRAF6	−1.595	1.93E-04
		c-fos	2.186	0.024
TWEAK signaling				
	CD8 T cells	TNFRSF12A	−2.436	0.003
		NIK	−1.833	3.59E-04
	NK cells	TNFRSF12A	2.409	0.001
		NIK	−1.574	0.002

**Fig. 6:**
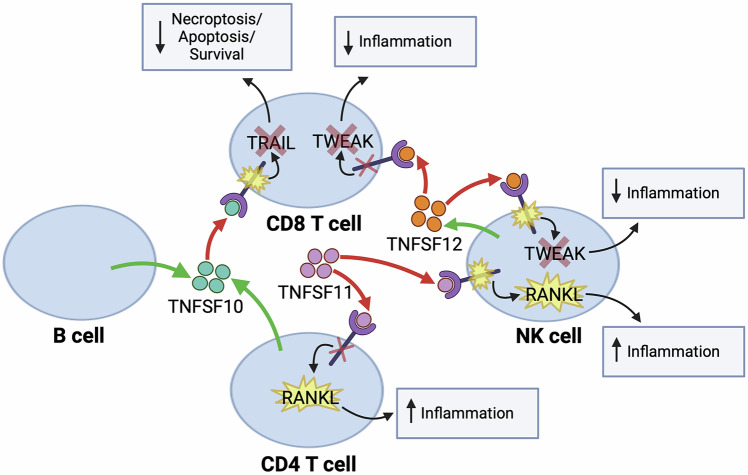
A schematic diagram summarizing our findings for cell-type specific dysregulation of TRAIL, RANKL, and TWEAK signaling pathways in ASD. Our study demonstrates upregulated levels of TNFSF10, TNFSF11, and TNFSF12 in the circulating plasma in ASD, with B cells, CD4 T cells, and NK cells likely contributing to these upregulations. In CD8 T cells, TRAIL and TWEAK signaling pathways are inhibited, ultimately leading to impaired cell fate regulation and a reduced inflammatory response. Although RANKL signaling is induced in both NK cells and CD4 T cells leading to an enhanced inflammatory response, the TWEAK signaling pathway is disrupted in NK cells resulting in inhibited inflammation in another population of this cell type. The green arrow indicates cell types producing a specific ligand, while the red arrow denotes the proposed signaling effect on their specific receptor.

Meanwhile, RANKL signaling was specifically dysregulated in CD4 T and NK cells, as indicated by impaired expression of TNFRSF11A, TRAF6, and c-fos in the ASD vs. control group (Table 2 and Fig. 5C). Finally, we observed significant upregulation of TNFSF12 expression in NK cells, suggesting this cell type as a potential source for the upregulated plasma levels of TNFSF12 in ASD (Table 2 and Fig. 5B). Additionally, TWEAK signaling molecules, including TNFRSF12A and NIK, were particularly disrupted in CD8 T cells and NK cells from individuals with ASD (Table 2 and Fig. 5D). We have included a schematic diagram in Fig. 6 to summarize the study results.

## Discussion

Previous studies have strongly indicated dysregulated immune phenotypes in the peripheral blood of subjects with ASD; however, the mechanisms behind this dysfunction remain unclear. In this study, we performed a high-throughput transcriptomic profiling of hundreds of immune genes in circulating immune cells from children with ASD. Interestingly, we identified differential expression of 50 immune genes and validated these gene signatures in independent studies on blood and brain tissues from individuals with ASD. These findings suggest the presence of an altered immune phenotype in our study cohort and support the relevance of these genes to ASD pathophysiology. Additionally, we explored the functional role of these gene signatures and determined that they are involved in cell activation and immune responses, including the TNF signaling pathway. Remarkably, plasma proteomics and single-cell RNA-seq analyses provided further insights into the molecular mechanisms underlying disrupted TNF signaling in ASD. We identified upregulated levels of TNFSF10, TNFSF11, and TNFSF12 in circulating plasma in ASD, with B cells, CD4 T cells, and NK cells potentially contributing to these upregulations. Furthermore, we identified dysregulated TRAIL, RANKL, and TWEAK signaling pathways in specific immune cells, including CD8 T cells, CD4 T cells, and NK cells. These observations provide novel insights into the molecular mechanisms of disrupted immune signaling in ASD.

In this study, we used an immune exhaustion panel to perform a targeted transcriptomic analysis of immune-related genes in circulating PBMCs from individuals with ASD. We identified 50 differentially expressed genes, 46 of which were downregulated and 4 were upregulated in children with ASD compared to controls. Notably, we observed downregulated expression of IL-6 in ASD; however, other studies have reported both increased and decreased IL-6 expression in ASD [10, 12, 37–39]. This discrepancy may be attributed to differences across studies in sample type (e.g., plasma, serum, or immune cells), analytical methods, and cohort characteristics, such as age, sex, and ethnicity [40–42]. Furthermore, we validated a total of 41 of our identified genes in independent ASD studies on blood and brain tissues as curated in BSCE platform [29–34]. Remarkably, most of the gene signatures were positively correlated in the blood and brain tissues in ASD. This finding is of particular interest as it suggests that immune changes in periphery are reflected in brain tissues in ASD. Additionally, we found significant correlations between ASD severity and the expression levels of three genes, namely JAK3, CUL2, and CARD11. Interestingly, we searched the Simons Foundation Autism Research Initiative (SFARI) online database (https://gene.sfari.org), and we found that CARD11 and CUL2-related family members (CUL1 and CUL7) are strong candidate genes for ASD. Meanwhile, JAK3 is a member of the JAK family of signaling molecules, and previous studies have shown dysregulated levels of another family member, JAK1, in the circulating immune cells of children with ASD as well in brain tissues of a mouse model of the disorder [43–45]. Together, we report gene signature changes that were similarly altered in circulating blood and brain tissues of individuals with ASD and suggest a potential relevance of immune dysregulation to ASD pathophysiology.

We then performed a multi-omics approach to determine what specific immune signaling pathways are disrupted in ASD at the transcriptomics, proteomics, and single-cell level. Although we observed an overall increase in the percent of CD4 T helper (Th) cells in ASD, this increase did not reach statistical significance. Previous studies have reported phenotypic alterations in CD4 subpopulations, including Th1, Th2, Th17, and regulatory T (Treg) cells, in individuals with ASD [16, 46, 47]. These findings underscore the need for further research to elucidate the molecular mechanisms contributing to imbalances in CD4 T cell subsets in ASD. Overall, we found reduced inflammation scores in cell-type specific immune cells, including B cells, NK cells, and T cell subsets, demonstrating the presence of disrupted immune function in ASD. Notably, we identified downregulation of several pathways in ASD related to lymphocyte activation, immune responses, and cell surface receptor signaling pathways, including TNF signaling. Additionally, we observed upregulated plasma levels of TNFSF10–12, suggesting dysregulated TNF signaling in ASD, specifically through TRAIL, RANKL, and TWEAK signaling pathways. Interestingly, previous studies demonstrated an upregulated level of TWEAK-related TNFSF12 in the serum from children with ASD [48, 49]. Although we detected an overall dysregulated immune phenotype in ASD, we observed a difference in the direction of change when comparing transcriptomic profiles of circulating immune cells to proteomic profiles in plasma samples. This warrants further investigation to determine the dynamic changes and crosstalk between immune compartments in ASD. Taken together, these findings suggest disrupted TNF-related signaling via TRAIL, RANKL, and TWEAK pathways in specific immune cell types in ASD that we have further investigated in this study.

Next, we sought to determine the molecular mechanisms underlying disrupted TRAIL signaling in circulating immune cells in ASD. TRAIL signaling is initiated by TNFSF10 binding to its TNFRSF10A receptor, which recruits the adaptor protein FADD [50, 51]. We found upregulated TNFSF10 expression in circulating plasma, B cells, and CD4 T cells in ASD, suggesting that these immune cells contribute to elevated plasma levels of TNFSF10. Additionally, we observed upregulated expression of TNFRSF10A and FADD specifically in CD8 T cells, indicating an induced ligand-receptor interaction in TRAIL signaling in ASD. Furthermore, FADD activation leads to caspase 8 (Casp8) activation, which can affect apoptosis, necroptosis, or cell survival, depending on the context [50–53]. For example, activated Casp8 indirectly leads to apoptosis through activation of effector caspases-3, -6 and -7 or it can inhibit necroptosis through cleaving a protein kinase complex consisting of RIPK1, RIPK3, and MLKL [50–53]. Here, we identified reduced expression of Casp8, RIPK1, RIPK3, and MLKL in CD8 T cells from individuals with ASD, suggesting inhibited apoptosis and necroptosis. Meanwhile, activated Casp8 could also promote cellular survival through activation of a signaling complex consisting of c-FLIP, TRADD, and IKBKG, which induces the expression of pro-survival genes via the AKT3 and MAPKs signaling cascades [50, 52, 53]. Apart from TRADD expression, we detected reduced expression of the survival-associated molecules, suggesting impaired survival of CD8 T cells in ASD. Interestingly, a previous study demonstrated that mice lacking Casp8 exhibited autism-like behaviors, suggesting a functional role of Casp8 in ASD pathophysiology [54]. Another study reported an association between a genetic mutation in AKT3 and ASD [55]. Overall, we report dysregulated TRAIL signaling in CD8 T cells, highlighting potential mechanisms for disrupted cell fate and immune responses in this cell type in ASD.

Furthermore, we identified dysregulated RANKL and TWEAK signaling pathways in NK cells and T cell subsets in ASD. RANKL signaling is triggered by the binding of TNFSF11 to its TNFRSF11A receptor, which induces the recruitment of TRAF6 adaptor protein that activates c-fos, a key transcription factor involved in osteoclastogenesis and inflammation [56, 57]. In this study, we found upregulated TNFRSF11 levels in the circulating plasma and dysregulated expression of TNFRSF11A in CD4 T cells and NK cells in ASD. Despite inhibited TRAF6 expression, we found upregulated expression of c-fos, suggesting an enhanced inflammatory response in these cell types in ASD. Meanwhile, TWEAK signaling is initiated by TNFSF12 binding to its receptor TNFRSF12A that indirectly leads to the activation of NFkB-inducing kinase (NIK), a critical downstream effector involved in tissue repair and inflammatory responses [58, 59]. Here, we observed upregulated levels of circulating TNFSF12 in ASD as previously reported [48, 49]. Additionally, we found upregulated TNFSF12 expression in NK cells, suggesting this cell type as an immune source for TNFSF12 in ASD. Furthermore, we found dysregulated expression of its TNFRSF12A receptor in NK cells and CD8 T cells in ASD, suggesting a dysregulated ligand-receptor signaling in TWEAK pathway. Meanwhile, we found reduced expression of NIK in both cell types indicating a disrupted downstream signaling in TWEAK pathway, which may result in impaired tissue repair and inflammatory responses in ASD. Together, these findings suggest disrupted RANKL and TWEAK signaling in NK cells and T cell subsets, providing novel insights into the mechanisms underlying immune dysregulation in these cell types in ASD.

Our study has several strengths. We recruited a well-defined study cohort that was matched in terms of age, sex, ethnicity, and other demographic characteristics. We focused on the age group of 2–4 years to identify molecular immune changes in ASD at an early stage of development, when most ASD symptoms begin to appear. Additionally, we adopted a holistic approach by integrating transcriptomics, proteomics, and single-cell RNA data analyses from the same study cohort. This approach revealed novel findings of dysregulated immune signaling pathways in ASD, from general circulating immune cells and molecules to the cell-type specific level in ASD. These observations could lead to future follow-up studies to investigate the underlying mechanisms of these dysregulations, which may lead to identifying potential therapeutic targets for ASD.

Nevertheless, our study has some limitations. We did not perform sex-specific analyses due to the limited sample size; however, future studies should determine whether our reported findings in ASD are linked to sex-specific factors. Additionally, we excluded subjects with immune conditions, such as asthma and allergies, to minimize confounding factors that could complicate the interpretation of our results. However, future studies should include subgroups of ASD and control subjects with and without immune conditions to further investigate the impact of these immune conditions on gene expression signatures and determine whether these factors have a more profound effect on individuals with ASD compared to the control group. Another limitation of our study is its cross-sectional design. Although we carefully selected subjects without confounding immune conditions, these subjects may not have exhibited these immune conditions at the time of study enrolment. Future studies should consider longitudinal analyses to more accurately capture dynamic changes in immune-related gene expression profiles in individuals with ASD and correlate these time-dependent changes with clinical and behavioral phenotypes in ASD.

In conclusion, our work supports previous reports of dysregulated immune phenotypes in ASD. We identified differential expression of 50 immune genes in circulating immune cells from individuals with ASD. Additionally, we found negative correlations between the expression of JAK3, CUL2, and CARD11 genes and the clinical severity of ASD symptoms. We validated these gene signatures in independent studies using blood and brain tissues from individuals with ASD, suggesting that altered immune profiles in the periphery are reflected in brain tissues in ASD. Furthermore, our multi-omics approach revealed altered TNF-related signaling in circulating immune cells in ASD, particularly in the TRAIL, RANKL, and TWEAK pathways. These signaling pathways were specifically dysregulated in NK and T cell subsets. Together, our study highlights potential mechanisms underlying immune dysregulation in ASD at the single-cell level. Future studies are needed to further elucidate the functional contribution of these dysregulated immune pathways to ASD pathophysiology.

## Supplementary information

Supplementary Figures 1–3

Supplementary Table 1: nCounter Immune Exhausion Panel

Supplementary Table 2: List of differentially expressed genes in the PBMCs of children with ASD

Supplementary Table 3: Correlation between our dataset and independent studies in ASD

Supplementary Table 4: Enriched pathways in PBMCs of children with ASD

Supplementary Table 5: List of differentially expressed genes at the single-cell level in ASD

Supplementary Table 6: List of differentially expressed proteins in plasma samples in ASD

## Data Availability

The raw datasets from this study will be available upon reasonable request to the corresponding author.

## References

[1] World Health Organization. Autism. 2023 [updated 15 November 2022]. Available from: https://www.who.int/news-room/fact-sheets/detail/autism-spectrum-disorders.

[2] Bjorklund G, Saad K, Chirumbolo S, Kern JK, Geier DA, Geier MR, et al. Immune dysfunction and neuroinflammation in autism spectrum disorder. Acta Neurobiol Exp (Wars). 2016;76:257–68. 28094817 10.21307/ane-2017-025

[3] Estes ML, McAllister AK. Immune mediators in the brain and peripheral tissues in autism spectrum disorder. Nat Rev Neurosci. 2015;16:469–86. 26189694 10.1038/nrn3978 PMC5650494

[4] McAllister AK. Immune contributions to cause and effect in autism spectrum disorder. Biol Psychiatry. 2017;81:380–2. 28137373 10.1016/j.biopsych.2016.12.024 PMC5650493

[5] Goines PE, Ashwood P. Cytokine dysregulation in autism spectrum disorders (ASD): possible role of the environment. Neurotoxicol Teratol. 2013;36:67–81. 22918031 10.1016/j.ntt.2012.07.006 PMC3554862

[6] Nour-Eldine W, Ltaief SM, Abdul Manaph NP, Al-Shammari AR. In search of immune cellular sources of abnormal cytokines in the blood in autism spectrum disorder: a systematic review of case-control studies. Front Immunol. 2022;13:950275. 36268027 10.3389/fimmu.2022.950275 PMC9578337

[7] Gładysz D, Krzywdzińska A, Hozyasz KK. Immune abnormalities in autism spectrum disorder-could they hold promise for causative treatment?. Mol Neurobiol. 2018;55:6387–435. 29307081 10.1007/s12035-017-0822-x PMC6061181

[8] Onore C, Careaga M, Ashwood P. The role of immune dysfunction in the pathophysiology of autism. Brain Behav Immun. 2012;26:383–92. 21906670 10.1016/j.bbi.2011.08.007 PMC3418145

[9] Tye C, Runicles AK, Whitehouse AJO, Alvares GA. Characterizing the interplay between autism spectrum disorder and comorbid medical conditions: an integrative review. Front Psychiatry. 2018;9:751. 30733689 10.3389/fpsyt.2018.00751 PMC6354568

[10] Ashwood P, Krakowiak P, Hertz-Picciotto I, Hansen R, Pessah I, Van de Water J. Elevated plasma cytokines in autism spectrum disorders provide evidence of immune dysfunction and are associated with impaired behavioral outcome. Brain Behav Immun. 2011;25:40–5. 20705131 10.1016/j.bbi.2010.08.003 PMC2991432

[11] Ashwood P, Krakowiak P, Hertz-Picciotto I, Hansen R, Pessah IN, Van de Water J. Associations of impaired behaviors with elevated plasma chemokines in autism spectrum disorders. J Neuroimmunol. 2011;232:196–9. 21095018 10.1016/j.jneuroim.2010.10.025 PMC3053074

[12] Enstrom AM, Onore CE, Van de Water JA, Ashwood P. Differential monocyte responses to TLR ligands in children with autism spectrum disorders. Brain Behav Immun. 2010;24:64–71. 19666104 10.1016/j.bbi.2009.08.001 PMC3014091

[13] Saad K, Abdallah AM, Abdel-Rahman AA, Al-Atram AA, Abdel-Raheem YF, Gad EF, et al. Polymorphism of interleukin-1β and interleukin-1 receptor antagonist genes in children with autism spectrum disorders. Prog Neuropsychopharmacol Biol Psychiatry. 2020;103:109999. 32526258 10.1016/j.pnpbp.2020.109999

[14] Xie J, Huang L, Li X, Li H, Zhou Y, Zhu H, et al. Immunological cytokine profiling identifies TNF-α as a key molecule dysregulated in autistic children. Oncotarget. 2017;8:82390–8. 29137272 10.18632/oncotarget.19326 PMC5669898

[15] Ashwood P, Krakowiak P, Hertz-Picciotto I, Hansen R, Pessah IN, Van de Water J. Altered T cell responses in children with autism. Brain Behav Immun. 2011;25:840–9. 20833247 10.1016/j.bbi.2010.09.002 PMC3039713

[16] Ellul P, Rosenzwajg M, Peyre H, Fourcade G, Mariotti-Ferrandiz E, Trebossen V, et al. Regulatory T lymphocytes/Th17 lymphocytes imbalance in autism spectrum disorders: evidence from a meta-analysis. Mol Autism. 2021;12:68. 34641964 10.1186/s13229-021-00472-4 PMC8507168

[17] Hughes HK, Onore CE, Careaga M, Rogers SJ, Ashwood P. Increased monocyte production of IL-6 after toll-like receptor activation in children with autism spectrum disorder (ASD) is associated with repetitive and restricted behaviors. Brain Sci. 2022;12. 10.3390/brainsci12020220 PMC8870658 35203983

[18] Jyonouchi H, Geng L. Associations between monocyte and T cell cytokine profiles in autism spectrum disorders: effects of dysregulated innate immune responses on adaptive responses to recall antigens in a subset of ASD children. Int J Mol Sci. 2019;20. 10.3390/ijms20194731 PMC6801811 31554204

[19] Bennabi M, Tarantino N, Gaman A, Scheid I, Krishnamoorthy R, Debre P, et al. Persistence of dysfunctional natural killer cells in adults with high-functioning autism spectrum disorders: stigma/consequence of unresolved early infectious events?. Mol Autism. 2019;10:22. 31123562 10.1186/s13229-019-0269-1 PMC6521549

[20] Gottfried C, Bambini-Junior V, Francis F, Riesgo R, Savino W. The impact of neuroimmune alterations in autism spectrum disorder. Front Psychiatry. 2015;6:121. 26441683 10.3389/fpsyt.2015.00121 PMC4563148

[21] Siniscalco D, Schultz S, Brigida AL, Antonucci N. Inflammation and neuro-immune dysregulations in autism spectrum disorders. Pharmaceuticals (Basel). 2018;11. 10.3390/ph11020056 PMC6027314 29867038

[22] Nour-Eldine W, Manaph NPA, Ltaief SM, Abdel Aati N, Mansoori MH, Al Abdulla S, et al. Discovery of a novel cytokine signature for the diagnosis of autism spectrum disorder in young Arab children in Qatar. Front Psychiatry. 2024;15:1333534. 38414501 10.3389/fpsyt.2024.1333534 PMC10896998

[23] Ltaief SM, Nour-Eldine W, Manaph NPA, Tan TM, Anuar ND, Bensmail I, et al. Dysregulated plasma autoantibodies are associated with B cell dysfunction in young Arab children with autism spectrum disorder in Qatar. Autism Res. 2024;17:1974–93. 39315457 10.1002/aur.3235

[24] Hao Y, Hao S, Andersen-Nissen E, Mauck WM 3rd, Zheng S, Butler A, et al. Integrated analysis of multimodal single-cell data. Cell. 2021;184:3573–87.e29. 34062119 10.1016/j.cell.2021.04.048 PMC8238499

[25] McGinnis CS, Murrow LM, Gartner ZJ. DoubletFinder: doublet detection in single-cell RNA sequencing data using artificial nearest neighbors. Cell Syst. 2019;8:329–37 e4. 30954475 10.1016/j.cels.2019.03.003 PMC6853612

[26] Ge SX, Son EW, Yao R. iDEP: an integrated web application for differential expression and pathway analysis of RNA-Seq data. BMC Bioinforma. 2018;19:534. 10.1186/s12859-018-2486-6 PMC6299935 30567491

[27] Chang J-G, Tu S-J, Huang C-M, Chen Y-C, Chiang H-S, Lee Y-T, et al. Single-cell RNA sequencing of immune cells in patients with acute gout. Sci Rep. 2022;12:22130. 36550178 10.1038/s41598-022-25871-2 PMC9772586

[28] Liberzon A, Birger C, Thorvaldsdottir H, Ghandi M, Mesirov JP, Tamayo P. The Molecular Signatures Database (MSigDB) hallmark gene set collection. Cell Syst. 2015;1:417–25. 26771021 10.1016/j.cels.2015.12.004 PMC4707969

[29] Kaya N, Colak D, Albakheet A, Al-Owain M, Abu-Dheim N, Al-Younes B, et al. A novel X-linked disorder with developmental delay and autistic features. Ann Neurol. 2012;71:498–508. 22213401 10.1002/ana.22673

[30] Alter MD, Kharkar R, Ramsey KE, Craig DW, Melmed RD, Grebe TA, et al. Autism and increased paternal age related changes in global levels of gene expression regulation. PLoS One. 2011;6:e16715. 21379579 10.1371/journal.pone.0016715 PMC3040743

[31] Chow ML, Li HR, Winn ME, April C, Barnes CC, Wynshaw-Boris A, et al. Genome-wide expression assay comparison across frozen and fixed postmortem brain tissue samples. BMC Genomics. 2011;12:449. 21906392 10.1186/1471-2164-12-449 PMC3179967

[32] Lanz TA, Guilmette E, Gosink MM, Fischer JE, Fitzgerald LW, Stephenson DT, et al. Transcriptomic analysis of genetically defined autism candidate genes reveals common mechanisms of action. Mol Autism. 2013;4:45. 24238429 10.1186/2040-2392-4-45 PMC4176301

[33] Sgadò P, Provenzano G, Dassi E, Adami V, Zunino G, Genovesi S, et al. Transcriptome profiling in engrailed-2 mutant mice reveals common molecular pathways associated with autism spectrum disorders. Mol Autism. 2013;4:51. 24355397 10.1186/2040-2392-4-51 PMC3896729

[34] Kong SW, Sahin M, Collins CD, Wertz MH, Campbell MG, Leech JD, et al. Divergent dysregulation of gene expression in murine models of fragile X syndrome and tuberous sclerosis. Mol Autism. 2014;5:16. 24564913 10.1186/2040-2392-5-16 PMC3940253

[35] Liu N, Jiang C, Cai P, Shen Z, Sun W, Xu H, et al. Single-cell analysis of COVID-19, sepsis, and HIV infection reveals hyperinflammatory and immunosuppressive signatures in monocytes. Cell Rep. 2021;37:109793. 34587478 10.1016/j.celrep.2021.109793 PMC8445774

[36] Sam SA, Teel J, Tegge AN, Bharadwaj A, Murali TM. XTalkDB: a database of signaling pathway crosstalk. Nucleic Acids Res. 2017;45:D432–D9. 27899583 10.1093/nar/gkw1037 PMC5210533

[37] El-Ansary A, Al-Ayadhi L. GABAergic/glutamatergic imbalance relative to excessive neuroinflammation in autism spectrum disorders. J Neuroinflamm. 2014;11:189. 10.1186/s12974-014-0189-0 PMC4243332 25407263

[38] Manzardo AM, Henkhaus R, Dhillon S, Butler MG. Plasma cytokine levels in children with autistic disorder and unrelated siblings. Int J Dev Neurosci. 2012;30:121–7. 22197967 10.1016/j.ijdevneu.2011.12.003 PMC6675569

[39] Rose DR, Yang H, Serena G, Sturgeon C, Ma B, Careaga M, et al. Differential immune responses and microbiota profiles in children with autism spectrum disorders and co-morbid gastrointestinal symptoms. Brain Behav Immun. 2018;70:354–68. 29571898 10.1016/j.bbi.2018.03.025 PMC5953830

[40] Decker ML, Grobusch MP, Ritz N. Influence of age and other factors on cytokine expression profiles in healthy children-A systematic review. Front Pediatr. 2017;5:255. 29312902 10.3389/fped.2017.00255 PMC5735141

[41] Miko, Poto A, Matrai P L, Hegyi P, Furedi N, Garami A, et al. Gender difference in the effects of interleukin-6 on grip strength—a systematic review and meta-analysis. BMC Geriatr. 2018;18:107. 29739343 10.1186/s12877-018-0798-z PMC5941705

[42] Chapman BP, Khan A, Harper M, Stockman D, Fiscella K, Walton J, et al. Gender, race/ethnicity, personality, and interleukin-6 in urban primary care patients. Brain Behav Immun. 2009;23:636–42. 19162168 10.1016/j.bbi.2008.12.009 PMC2694851

[43] Ahmad SF, Nadeem A, Ansari MA, Bakheet SA, Al-Ayadhi LY, Attia SM. Upregulation of IL-9 and JAK-STAT signaling pathway in children with autism. Prog Neuropsychopharmacol Biol Psychiatry. 2017;79:472–80. 28802860 10.1016/j.pnpbp.2017.08.002

[44] Ahmad SF, Ansari MA, Nadeem A, Bakheet SA, Alzahrani MZ, Alshammari MA, et al. Resveratrol attenuates pro-inflammatory cytokines and activation of JAK1-STAT3 in BTBR T(+) Itpr3(tf)/J autistic mice. Eur J Pharm. 2018;829:70–8. 10.1016/j.ejphar.2018.04.008 29654783

[45] Ahmad SF, Ansari MA, Nadeem A, Bakheet SA, Alsanea S, Al-Hosaini KA, et al. Inhibition of tyrosine kinase signaling by tyrphostin AG126 downregulates the IL-21/IL-21R and JAK/STAT pathway in the BTBR mouse model of autism. Neurotoxicology. 2020;77:1–11. 31811869 10.1016/j.neuro.2019.12.003

[46] Ahmad SF, Zoheir KM, Ansari MA, Nadeem A, Bakheet SA, Al-ayadhi LY, et al. Dysregulation of Th1, Th2, Th17, and T regulatory cell-related transcription factor signaling in children with autism. Mol Neurobiol. 2017;54:4390–400. 27344332 10.1007/s12035-016-9977-0

[47] Moreno RJ, Abu Amara R, Ashwood P. Toward a better understanding of T cell dysregulation in autism: an integrative review. Brain Behav Immun. 2025;123:1147–58. 39378971 10.1016/j.bbi.2024.10.009

[48] Artik A, Oztelcan Gunduz B, Mizrak S, Isik U. Increased serum levels of tumour necrosis factor-like weak inducer of apoptosis in children with autism spectrum disorder. Int J Dev Disabil. 2023;69:611–6. 37346259 10.1080/20473869.2022.2143033 PMC10281418

[49] Bilgiç A, Özgül Katırcıoğlu D, Taş MB, Kılınç İ, Oflaz MB, Akça ÖF Serum BAFF, APRIL, TWEAK, TNFSF18, TNFR2, and TNFRS12A levels in preschool children with autism spectrum disorder. Int J Dev Disabil. 1–9. 10.1080/20473869.2024.2359143 PMC13202648 42205937

[50] Tang D, Kang R, Berghe TV, Vandenabeele P, Kroemer G. The molecular machinery of regulated cell death. Cell Res. 2019;29:347–64. 30948788 10.1038/s41422-019-0164-5 PMC6796845

[51] Wajant H. TRAIL- and TNF-induced signaling complexes—so similar yet so different. EMBO J. 2017;36:1117–9. 28400401 10.15252/embj.201796997 PMC5412772

[52] Oberst A, Green DR. It cuts both ways: reconciling the dual roles of caspase 8 in cell death and survival. Nat Rev Mol Cell Biol. 2011;12:757–63. 22016059 10.1038/nrm3214 PMC3627210

[53] Refaat A, Abd-Rabou A, Reda A. TRAIL combinations: the new ‘trail’ for cancer therapy (Review). Oncol Lett. 2014;7:1327–32. 24765133 10.3892/ol.2014.1922 PMC3997674

[54] Suarez-Pereira I, Garcia-Dominguez I, Bravo L, Santiago M, Garcia-Revilla J, Espinosa-Oliva AM, et al. The absence of caspase-8 in the dopaminergic system leads to mild autism-like behavior. Front Cell Dev Biol. 2022;10:839715. 35493109 10.3389/fcell.2022.839715 PMC9045412

[55] Alcantara D, Timms AE, Gripp K, Baker L, Park K, Collins S, et al. Mutations of AKT3 are associated with a wide spectrum of developmental disorders including extreme megalencephaly. Brain. 2017;140:2610–22. 28969385 10.1093/brain/awx203 PMC6080423

[56] Li B, Wang P, Jiao J, Wei H, Xu W, Zhou P. Roles of the RANKL-RANK Axis in Immunity-Implications for Pathogenesis and Treatment of Bone Metastasis. Front Immunol. 2022;13:824117. 35386705 10.3389/fimmu.2022.824117 PMC8977491

[57] Takegahara N, Kim H, Choi Y. RANKL biology. Bone. 2022;159:116353. 35181574 10.1016/j.bone.2022.116353 PMC9035122

[58] Zhang Y, Zeng W, Xia Y. TWEAK/Fn14 axis is an important player in fibrosis. J Cell Physiol. 2021;236:3304–16. 33000480 10.1002/jcp.30089

[59] Burkly LC, Michaelson JS, Zheng TS. TWEAK/Fn14 pathway: an immunological switch for shaping tissue responses. Immunol Rev. 2011;244:99–114. 22017434 10.1111/j.1600-065X.2011.01054.x

