# Supplementary material for: A multi-omics approach reveals dysregulated TNF-related signaling pathways in circulating NK and T cell subsets of young children with autism
Source: Genes Immun. 2025 Jul 30;26(5):462–74. doi: 10.1038/s41435-025-00349-z (PMC12527906; doi:10.1038/s41435-025-00349-z)
Supplement: Supplementary file 1 — Supplementary Figures 1–3 [file 41435_2025_349_MOESM1_ESM.pdf]

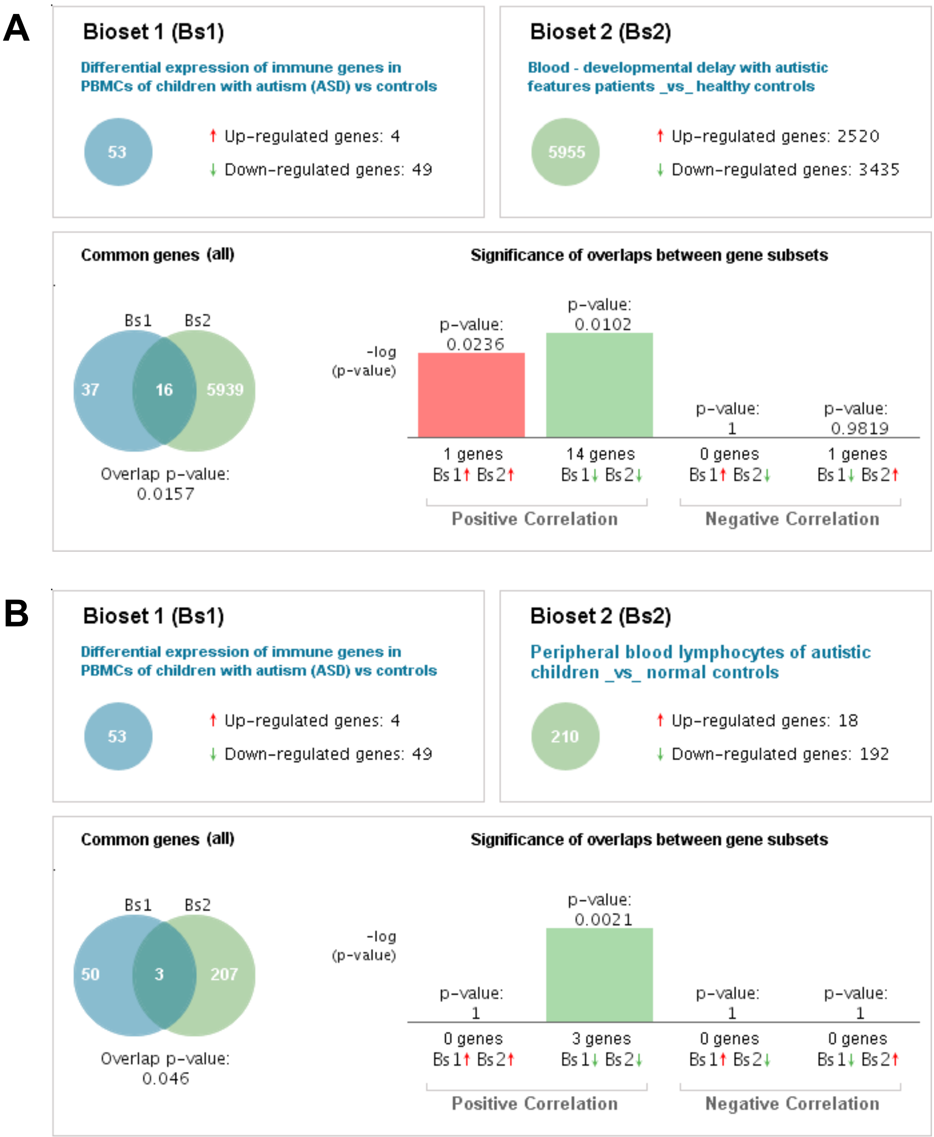

Supplementary Figure 1. BaseSpace Correlation Engine (BSCE) analysis validates our differentially expressed genes in independent studies on human blood samples in ASD. (A) Correlation analysis reveals 16 common markers between our dataset (Bioset 1) and independent study conducted on blood samples of subjects with developmental delay and autistic features vs. control group (Bioset 2). **(B)** Three markers are common between our dataset (Bioset 1) and independent study on lymphocytes of subjects with ASD vs. control group (Bioset 2).

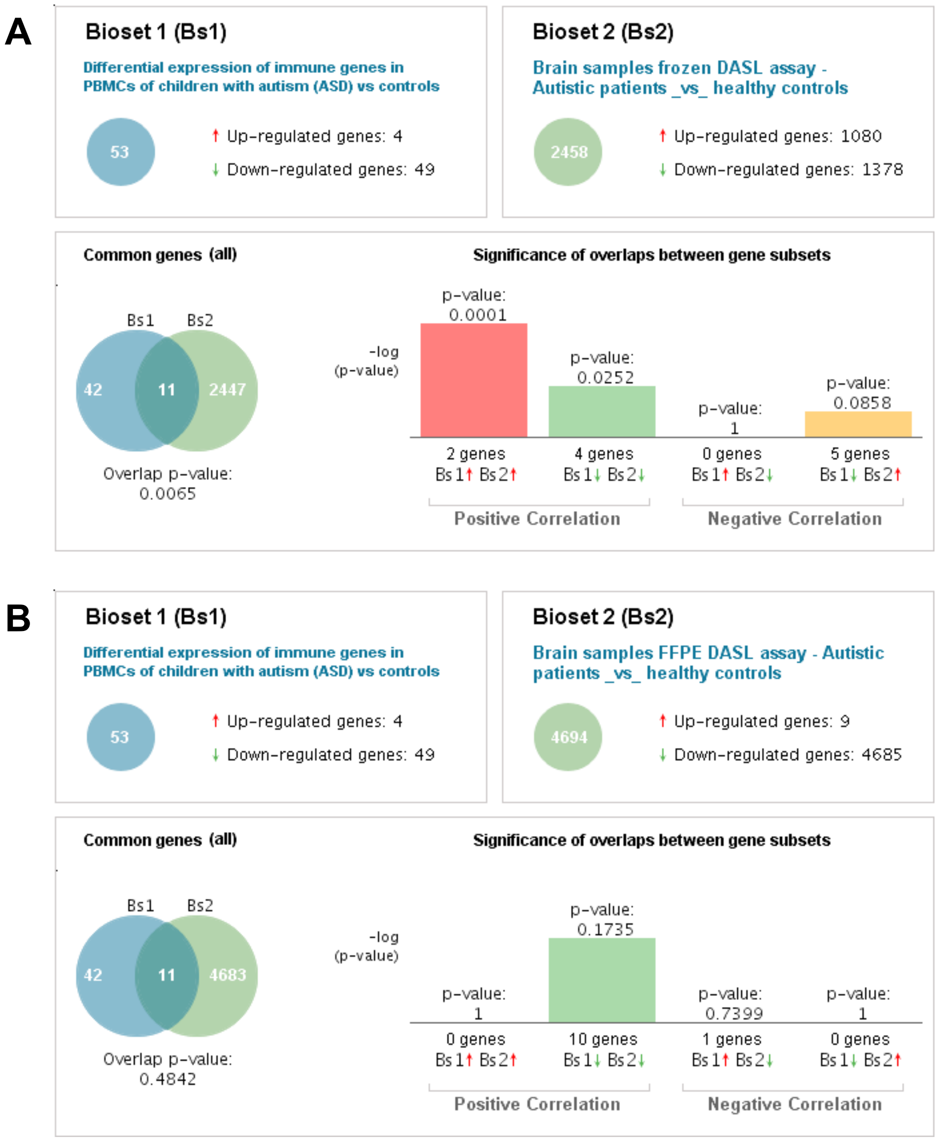

Supplementary Figure 2. BSCE analysis validates our differentially expressed genes in independent studies on human brain samples in ASD. (A–B) Correlation analysis shows 11 common markers between our dataset (Bioset 1) and independent study conducted on the prefrontal cortex of subjects with ASD in comparison to control group (Bioset 2).

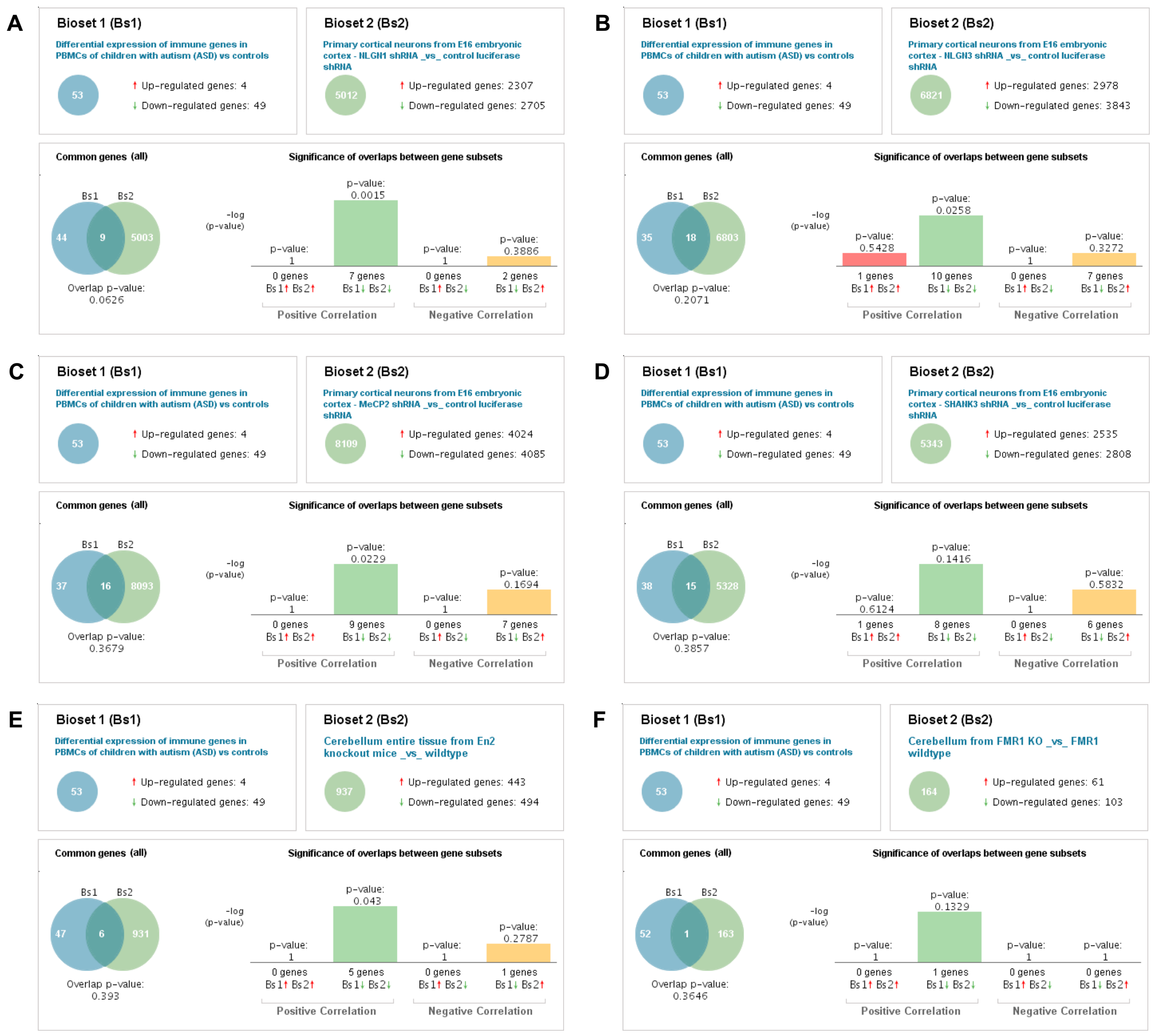

Supplementary Figure 3. BSCE analysis validates our differentially expressed genes in independent studies on brain samples of mouse models of ASD. (A–B) Correlation analysis reveals nine and eighteen common markers between our dataset (Bioset 1) and independent studies conducted on the primary cortical neurons with the knockdown of NLGN1 and NGL3, respectively, in comparison to the control group (Bioset 2). **(C–D)** Sixteen and fifteen markers are common between our dataset (Bioset 1) and independent studies on the cortical neurons with the knockdown of MeCP2 and SHANK3, respectively, comapred to the control group (Bioset 2). **(D)** Six and one markers are common between our dataset (Bioset 1) and independent studies on the cortical neurons with the knockdown of En2 and FMR1, respectively, in comparison to the control group (Bioset 2).
